# Reports of Cases

**Published:** 1853-06

**Authors:** Thos. Hall


ART. II.—*Reports of Cases*. By THOS HALL, M.D. Read before the Illinois State Med. Society, June, 1853.

THE first disorder of any consequence in Stark County after our annual meeting in last June, was mucous diarrhœa. It appeared in the same places and was characterized by the same appearances as the diarrhœa which preceded the dysentery of the past year. *Quinine*, which quickly and invariably cured the disorder in that year, produced no benefit in this. Turpentine, which has long been with me a favorite remedy in this form of diarrhœa, quickly checked and in a short time cured every case, although in the preceding year it produced little or no real benefit.

This disorder, as in the preceding year, was followed by dysentery, the cases limited in number and trifling in character compared with the previous epidemic. There was one feature in the cases worthy of note. The disease was located, in every instance, in the lowest portion of the colon or upper portion of the rectum—that is as near as we can judge on external examination—whereas in the preceding year it was as invariably seated in the left side of the arch, or upper portion of the descending colon. I commenced the treatment with the same remedies that I had found so efficacious before, as mentioned in my portion of the report on practical medicine, read at our last meeting, but without the least appreciable benefit. I confess having been disappointed; recollecting, however, that the treatment which was successful in controlling the diarrhœa of the past year, was with but slight modification equally successful in arresting the subsequent dysentery, Turpentine and opium were given with the happiest effect in every case, save two, and these yielded at once to iodide injections, which treatment I saw strongly recommended in some of the journals at the time they were under treatment. I had previously used acetate of lead in cold water, and turpentine in mucilage without any benefit. In one case the change was immediate, and one injection only was used in both, the patients expressing themselves as feeling much easier directly after its use. I tried it in a sporadic case some time afterwards, with the same happy result—on the whole I regarded this injection as being the best yet used in the treatment of this troublesome disease, and especially when it is so low in the bowels, that it can be safely, easily, and certainly reached.

Immediately following the dysentery came typhoid fever, which was more fatal than any other disease during our medical year. As everything connected with this disease must be interesting to the profession, I shall devote more space to it; at the same time I would urge on my professional brethren, when they have the opportunity, to observe it closely and, discarding all written opinions, endeavor to ascertain its cause; for until we can determine this matter, our practice will be as diversified as the various theories that are now held respecting its origin and nature. So far as I saw, and so far as I have made inquiry, there has been no fever in our section, except in houses under which there was a cellar, and food more or less kept in the cellar. In 1851 the cellars were generally filled with water from the excessive rains. In 1852, they were unusually damp, and I formed an opinion, whether right or wrong time alone will determine, that typhoid fever is caused by a something that passes through, or at least into the intestinal canal, and that in tainted food. This opinion I now hold, and shall certainly not give it up for the one last printed in the Chicago Med. Journal, "that hitherto remittent and continued fever (including typhus, typhoid, yellow fever, &c.,) have a common origin," for to me it appears there is and can be no more relationship existing betwixt typhoid and the other fevers, than betwixt a mild case of small pox and a severe case of itch. In past years, I had succeeded as well as any of my brethren in this form of fever, and my treatment had been as little medicine as possible, so that every obstacle to a favorable termination was removed, and unfavorable symptoms, as they arose, combated. The only remedy that I constantly used, and can unhesitatingly recommend in every form of low fever, was Hope, and this I gave in no sparing quantity. But, truthfully speaking, no course of medical treatment has been perfectly satisfactory to my mind—we have as yet no sure guide for unerring safe practice. The first cases of typhoid fever were in my own family—three were apparently impressed at the same time, at least they commenced complaining on the same morning; they ran the usual course under the usual treatment, and recovered—sick from twenty-four to thirty days; no alarming symptoms, except in one case. As I found turpentine the best remedy, when ulceration was threatening or had actually commenced, I determined, that should any more cases occur in my family, I would try its power in preventing ulceration of the intestines and intestinal and mesenteric glands, and give it from the beginning. The opportunity was soon afforded in a daughter of eight years old. I amused her with cold water and homœopathic treatment until there was unmistakable evidence of the disease.

The discharges from the bowels are, if carefully examined, sufficiently diagnostic. Their odor is peculiar, and if they are allowed to stand, there is a sediment that I cannot well describe, but beneath the general dark color of the fæces there is deposited something like dirty yellow and green paint. As soon as these characters were perceptible, I gave her ten drops of turpentine and three of laudanum, every four hours; in twelve hours there was visible improvement in her countenance, tongue, and character, and frequency of the intestinal discharges.

She took nothing except the above during her attack; the opium was omitted according to circumstances. Not a single symptom occurred, during the disease, creating any alarm, and yet its duration, from first complaining to anything like rapid convalescence, was twenty-two days. When awake, her mind was always clear; the appetite never entirely destroyed; her bowels acted from twice to four times in twenty-fours hours, but of no bad odor or color after the first day of taking the turpentine. In this, as in every case, whether mild or severe, male or female, the head became all but bald during convalescence.

The next case in which I tried the turpentine and opium, was in a young man, of 23 years. He first felt indisposed on a Saturday; I saw him on Monday and suspected the disorder, but amused him during the day with a semblance of medical treatment. The next morning at five o'clock, the fæces were typhoid, and were discharged every two or three hours in considerable quantity. I mixed two parts of turpentine with one of laudanum, and ordered thirty drops every four hours until I saw him again. I visited him a little after nine at night, and the change in his countenance for the better was very visible; his tongue was moist and almost clean. and his bowels had acted but once. Surprised somewhat at his improved condition, I was calculating the number of doses he had taken, when his nurse said "15, you ordered his medicine every hour and I have been very regular in giving it." I suspended the treatment until next day, and gave it three times in the twenty-four hours, lessening the quantity of laudanum and increasing that of the turpentine. On the eleventh day from his feeling indisposed I found him at the breakfast table with about a quarter of a healthy appetite, and he regularly rose to his meals afterward; but he did not appear to advance in his recovery from the eleventh to somewhere about the twenty-first day, and then convalescence was rapid; diarrhœa during this interval would threaten to return if the medicine was omitted for a whole day.

These two are a fair sample of all the cases treated with turpentine from the commencement; it certainly prevented the tendency to glandular disorganization, and, compared with the cases reported by Jenner, shortened their duration by some five or six days. For nourishment, I gave regularly Liebig's beef tea, and any gruel that the taste or caprice of the patient might crave.

In conclusion, I will merely observe that in no cases where this treatment was commenced by the fourth or fifth day did any alarming symptoms show themselves. Everything moved on with pleasure and satisfaction to myself, and with such safety and comparative comfort to the patients as to justify me in bringing the matter before the profession.

Puerperal fever has been rife, and I believe invariably fatal, in Stark and neighboring Counties. No treatment appeared to be of any real benefit. In one case where the suffering was unusually severe, I abstracted about 12 ounces of blood, the pain was not only materially lessened, but—cut short by at least some eight or ten hours. I should hesitate to recommend it in any case of epidemic puerperal peritonitis.

During the prevalence of this disease, there was scarcely a woman who had not much more than ordinary tenderness about the womb, accompanied with a greater or less degree of febrile disturbance. I refer to these cases principally to call the attention of the juniors to a symptom, if it may be so termed, of fearful import, which was present in every instance—an impression on the mind, in the earliest months of pregnancy, that they should die before or at the period of child-birth ; an impression that no argument could remove, and it was useless trying to lessen their despondency by the administration of hope. The prognostic value of this symptom has not been noticed by our systematic writers in the way that its importance demands.

According to my experience, (and in this *one* respect it extends over 40 years,) an impression of a fatal termination firmly fixed on the mind of a patient, during the incubation of disease, is a certain sign that it is incurable, and when fully developed, as a general rule rapidly fatal.

